# Experience of clinical incivility and stress in nursing students: A mixed-method study

**DOI:** 10.1371/journal.pone.0329333

**Published:** 2025-07-31

**Authors:** Youngjin Lee, Cheryl Brandt, Younglee Kim

**Affiliations:** 1 College of Nursing, Eulji University, Gyeonggi-do, Republic of Korea; 2 Department of Nursing, California State University San Bernardino, California, United States of America; University of Maribor, SLOVENIA

## Abstract

Nursing students often experience uncivil words or actions during clinical practice which can lead to significant stress. The purpose of our study was to quantitatively investigate the degree of clinical incivility and stress experienced by students and the statistical relationship between them and qualitatively explore students’ personal experiences of clinical incivility and stress. Our study was conducted from April 25 to May 27, 2023, using a mixed-method design. Phase One, the quantitative component, used a non-experimental, descriptive, cross-sectional study design with a 10-minute self-administered online survey. Phase Two, the qualitative component, used an in-person focus group to collect data on participants’ personal experiences of clinical incivility and stress. A total of 159 junior and senior pre-licensure nursing students attending a clinical practicum of a Bachelor of Science in Nursing (BSN) program in South Korea completed the quantitative online survey. Subsequently, 20 students voluntarily attended focus group sessions. To analyze the online survey data the study sample was divided into a clinical incivility group and a non-clinical incivility group. A group comparative analysis using chi-square and t-test was conducted. To analyze the qualitative focus group data, Colaizzi’s method to identify significant themes was used. Prevalence of clinical incivility experience in the students was 79.9% (n = 123 out of 159). Clinical incivility was significantly positively correlated with the students’ reported stress level (r = .317, p < .001); students who experienced greater clinical incivility reported higher levels of stress. Additionally, higher levels of clinical incivility were linked to lower levels of satisfaction with clinical practice and the clinical site. Analysis of the focus group data revealed four main themes regarding the students’ experiences: *1) Being defenselessly exposed to verbal or nonverbal abuse, 2) Not being treated as a student nurse, 3) Experiencing a combination of negative feelings, and 4) Taking incivility for granted in the clinical site.* Major perpetrators of clinical incivility among nursing students were nurses at the clinical sites. To prevent and manage clinical incivility, nursing schools and clinical agencies must collaborate to establish incivility monitoring, communication, and evidence-based policies and intervention programs.

## 1. Introduction

Incivility exists in various forms and aspects of human society [1,2]. It is related to the structure of society or the balance of power [3]. Incivility, prominent and widespread [4], is described as antisocial and deviant behavior with a rude and disrespectful intention to harm others by bullying or verbal abuse [5–7]. According to the Merriam-Webster online dictionary, incivility is also defined as “the state of being uncivil, marked by rude or discourteous behavior” [8]. Parse stated [9], “incivility is blatant disregard of others, a violation of human dignity” (p. 261).

Uncivil actions such as neglect, scolding, harsh language, belittling remarks, sarcasm, gossiping, harassment, physical attacks, and sexual harassment negatively affects mental health [10] resulting in stress, depression, anxiety, and burnout [11–13]. Such negative and violent words or actions lacking respect and consideration for others can cause physical, psychological, or emotional damage [5] and damage relationships [14].

The impact of incivility varies significantly across cultural and institutional contexts. Pearson and Porath (2005) found that in the United States and Canada, workplace incivility adversely affects job performance and organizational commitment, leading to increased burnout and stress among employees [15]. By contrast, research in Asia reveals how hierarchy and cultural norms shape experiences of incivility. For example, Curhan, et al. found that in Japan, workplace hierarchy can lead to subtle forms of incivility that, while not overt, still impact employee well-being [16]. Similarly, Moon and Sanchez-Rodriguez reported that in South Korea, cultural expectations around respect and authority influence how incivility is perceived and addressed in both educational and professional settings [17]. This dynamic is reflected in Asian workplace cultures where superiors frequently assign unpleasant or troublesome tasks to subordinates without considering their dignity [18]. Addressing incivility is essential for fostering a healthier and more supportive workplace environment [19].

Incivility is also rising in higher education institutions [20], seen in classrooms between faculty and students [21–24] and clinical areas between students and staff nurses [25–27]. Such incivility disrupts the teaching-learning process [28] and undermines the morale of both students and staff [29].

Clinical practicums, a mandatory component of the nursing curriculum, allow nursing students to experience nursing practice firsthand, enhancing their nursing knowledge and skills and contributing to professional identity development [26,30]. According to qualitative research reported by Hakojarvi et al., both verbal and non-verbal bullying from nursing staff during clinical rotations negatively affected healthcare students’ motivation and professional engagement in Finland [31]. Similarly, Smith et al. found undergraduate baccalaureate nursing students in the USA reported that bullying behaviors during clinical practice impeded their learning and led to increased physical and psychological harm [32]. Kim found that experiences of incivility during clinical practicums among Korean undergraduate nursing students were negatively correlated with their professional nursing values [30]. Shen observed that Chinese nursing students in operating room clinical practicums experienced incivility, describing the clinical learning environment as unhealthy, uncivil, and disrespectful [33]. Nursing students, especially vulnerable due to their relative lack of knowledge, skills, and experience, frequently encounter incivility from healthcare professionals including nurses as well as from patients and families [34]. This can reduce students’ learning, satisfaction with nursing programs [21], and confidence in becoming nurses [34].

Incivility can be a source of stress experienced during clinical practice [35]. According to WHO [36], stress is “a state of worry or mental tension caused by a difficult situation…stressful situations can cause or exacerbate mental health conditions… affecting daily functioning, including at work or school”. Stress often arises in response to rudeness, which is frequently linked to the breakdown of social norms and expectations [37]. When individuals experience uncivil behavior, they may view it as a violation of their dignity and respect, triggering a stress response that is further intensified by the ambiguity and unpredictability of such behavior, leaving them feeling vulnerable and powerless [38]. Incivility experience during clinical practicums was found to be a significant factor in increased stress levels of nursing students [39–41] which can lead to feelings of shame, anxiety, depression, and even thoughts of suicide [42]. Furthermore, chronic stress decreases confidence and self-esteem, hindering effective learning and academic performance [34].

More research on the relationship between clinical incivility and stress is needed. There are relatively few published mixed-methods studies of clinical incivility as it relates to stress in nursing students. Since the degree and perception of incivility and stress vary depending on sociocultural norms and values [43], collecting quantitative data alone may limit nuanced understanding of students’ experiences of clinical incivility and stress. Similarly, employing only qualitative research designs may introduce methodological limitations such as sampling bias and respondent bias due to researcher presence [44]. In contrast, a mixed-methods approach permits a comprehensive view by integrating both qualitative and quantitative data, capturing trends and insights [45]. This method enhances validity through triangulation which corroborates results and strengthens conclusions [46], offering rich, detailed data by combining broad quantitative characteristics with in-depth qualitative context [47]. Therefore, a mixed-methods research design can potentially yield a deeper understanding of students’ experience.

The purpose of our mixed-methods study was twofold: To investigate the degrees of nursing students’ experience of clinical incivility and stress and the association between them, and to explore the students’ personal experiences of clinical incivility related to stress. Specific research questions for the quantitative component were:

1. What is the prevalence of clinical incivility among nursing students?
2. How does the incivility experienced by nursing students during their clinical practicum relate to the stress they felt?

The research question for the qualitative component was:

1. How do nursing students describe their experiences of incivility by nurses during clinical practice?

## 2. Methods

### 2.1. Study design and procedure

This study employed a mixed-methods research design in two phases from April 25, 2023, to May 27, 2023. Phase One, the quantitative component, used a non-experimental, descriptive, cross-sectional study design with a 10-minute self-administered online survey. Phase Two, the qualitative component, used an in-person focus group to collect data on participants’ personal experiences of clinical incivility and stress.

For Phase One, one primary investigator, who was not affiliated with the students, sent an email invitation to join the study to all junior and senior nursing students in the Bachelor of Science in Nursing (BSN) program. The email included the study information, an online written survey participation consent form, and the survey link. Participation was voluntary with survey completion occurring after the online consent form was completed. Upon survey completion, each participant received a $5 electronic gift card.

In Phase Two, the same primary investigator emailed the students who completed the online survey inviting participants with clinical incivility experiences to attend an in-person focus group. Following a literature review about clinical incivility and stress, focus group interview questions were developed and piloted with nursing students. Three questions were used for the focus group interviews:

1. How was your experience with nurses during your clinical practice?
2. How did you react when you experienced incivility during your clinical practice?
3. What do you think is the cause of incivility during clinical practice?

A total of three in-person focus group sessions were conducted on campus by two primary investigators, both PhD-prepared female nursing faculty members with no prior personal contact or relationship with the participating students. These investigators, who specialized in research related to BSN students, collaborated to develop the procedures and methods for each session based on their experience and findings in the literature review before jointly conducting the sessions. Prior to the sessions, participants completed written consent forms, including permission for audio recording. Each focus group, consisting of three to nine nursing students who volunteered to participate, lasted approximately one hour. To ensure all participants had a clear understanding, the investigators first explained the key terms and concepts related to incivility as identified in the literature review. Sessions were audio-recorded and field notes from each focus group were made by the same two primary investigators. At the end of the focus group session, each participant received a $10 gift card. After each focus group, the primary investigators discussed data saturation for the focus group sessions.

### 2.2. Participants

Study participants were all nursing students in the BSN program at a university located in Gyeonggi-do, South Korea. Study inclusion criteria were a) 18 years of age or older, b) enrolled in the BSN program during the Academic Year (AY) 2023–2024, c) junior or senior nursing students with at least a month of clinical experience in hospital settings, and d) able to read and understand the Korean language. Exclusion criteria were self-report of mental illness or taking medicine prescribed by a healthcare provider for mental illness.

According to G*power 3.1.9.7 version calculations using an effect size of .3, alpha of .5, and power of .95, the required sample size was at least 112 participants. Ultimately 170 students completed the online survey for quantitative data; data from only 159 students were included after applying inclusion and exclusion criteria. Twenty nursing students voluntarily completed a Phase Two in-person focus group session.

### 2.3. Quantitative data instruments

The online survey included four questionnaires: a) participant characteristics, b) a Clinical Practice Evaluation used by the BSN program, c) a Korean version of the Uncivil Behavior in Clinical Nursing Education (K-UBCNE), and d) a Korean version of the Perceived Stress Scale (K-PSS).

The participant characteristics questionnaire included ten questions about age, marital status, education, employment, religion, Grade Point Average (GPA), clinical incivility experience from classmates, patients, clinical instructors, nurses, physicians, and other staff, mental illness history, and current psychiatric medication.

The BSN Clinical Practice Evaluation Survey was developed for program evaluation use by the College of Nursing. It includes three questions about BSN Program Satisfaction, Clinical Practice Satisfaction, and Clinical Site Satisfaction. A five-point Likert-type scale ranging from 1 = very dissatisfied to 5 = very satisfied is used.

The Uncivilized Behavior in Clinical Nursing Education (UBCNE) scale was developed by Anthony and Yastik in 2011 [48]. The initial UBCNE version included 20 items measuring the uncivil experiences of nursing students received from nurses in clinical learning settings. Anthony et al. [49] revised it to 12 items representing three subscales: Hostile/Mean (3 items), Exclusionary Behaviors (5 items), and Dismissive (4 items). Items use a 5-point Likert-type scale (0 = never to 4 = very often); possible scores range from 0-48.

Our study used the K-UBCNE modified by Jo and Oh in 2016 [50]. It has 13-items with three subscales: Exclusion (5 items), Contempt (5 items), and Refusal (3 items), and uses the same five-point Likert type scale (0 = never to 4 = very often). Possible scores range from 0-52. High scores again indicate more uncivil experiences.

Anthony et al. [50] reported Cronbach’s alpha coefficient scores of the UBCNE from.84 to .86. The K-UBCNE developed by Jo and Oh [50] demonstrated a Cronbach’s alpha score of .86, indicating good internal consistency. Kim et al. [35] reported Cronbach’s alpha scores of the K-UBCNE ranging from .78 to .88. Cronbach’s alpha coefficient scores of the K-UBCNE in our study ranged from .87 to .94.

Lastly, we used the Perceived Stress Scale (PSS) developed by Cohen and colleagues in 1983 [51] to quantify stress levels of nursing students. The initial version of the PSS included “14 items to measure the degree of stress perceived in daily life” [51]. In 1988, a 10-item version of the PSS (PSS-10) using a 5-point Likert-type scale by Cohen and Williamson was published [52]. Possible scores of the PSS-10 range from 0-40, with higher scores reflecting higher perceived stress levels. For our study, the Korean version of the PSS-10 (K-PSS-10), translated by Lee et al. [53] with the same 5-point Likert-type scale (0 = never to 4 = very often), was used. In Cohen et al.’s study [52] using a sample of American adults, the Cronbach’s alpha coefficient score of PSS-10 was.78. In the study by Lee et al. [53], the Cronbach’s alpha coefficient for the K-PSS-10 was .74, while in our study it was .75, both findings indicating acceptable reliability and supporting the instrument’s appropriateness for use in this study context.

### 2.4. Data analysis

SPSS Statistics 23 was used to analyze Phase One survey data; respondents’ anonymity was maintained. Descriptive statistics of sample characteristics and prevalence of clinical incivility among participants were calculated. The sample was subdivided into a Clinical Incivility group who experienced clinical incivility during their clinical practice and a No Clinical Incivility group who reported experiencing no clinical incivility. A t-test was run to compare the mean scores on the PSS-10 stress scale between the two groups. The chi-square test for categorical variables (i.e., marital status, education, employment, and religion) was calculated to determine between-group differences. Pearson correlations were calculated among age, GPA, total scores on the K-UBCNE, and the K-PSS, and scores on the BSN Program Satisfaction, Clinical Practice Satisfaction, and Clinical Site Satisfaction items in the BSN Clinical Practice Evaluation Survey.

Colaizzi’s method [54] was used to analyze focus group data. Data from three sessions with a total of 20 nursing students were analyzed. First, the research team carefully read the transcripts of focus group voice recordings several times to grasp the general sense of the data. Each researcher independently reviewed the transcripts line by line, highlighting significant words, phrases, and statements made in response to each question. These significant statements were then coded and grouped into meaningful categories using a coding tree structure. Through team discussions, these categories were further synthesized into broader themes that captured the essence of participants’ experiences. To enhance trustworthiness, the authors engaged in reflexivity by bracketing their own preconceptions and assumptions prior to and during analysis. An audit trail was maintained to document analytic decisions and theme development. Validation was strengthened through investigator triangulation, where each author independently analyzed the data and then met to compare, discuss, and reconcile discrepancies in coding and interpretation. This iterative process continued until consensus was achieved on all themes. Due to the focus group format and confidentiality protocols, member checking with participants was not conducted.

### 2.5. Ethical considerations

Our study’s Institutional Review Board (IRB) approval (EU23-03) was obtained from a university located in Gyeonggi-do, South Korea. All study participants completed informed written consent and voluntarily participated in the online survey for quantitative data collection. A smaller group of participants also attended in-person focus group sessions after the written consent forms. Informed consent outlined the study’s purpose, procedures, risks, and participants’ right to withdraw at any time without penalty. Participants were assured of confidentiality, informed about available counseling services, and advised of privacy protection and secure data handling practices.

## 3. Results

### 3.1. Quantitative data

**Fig 1:**
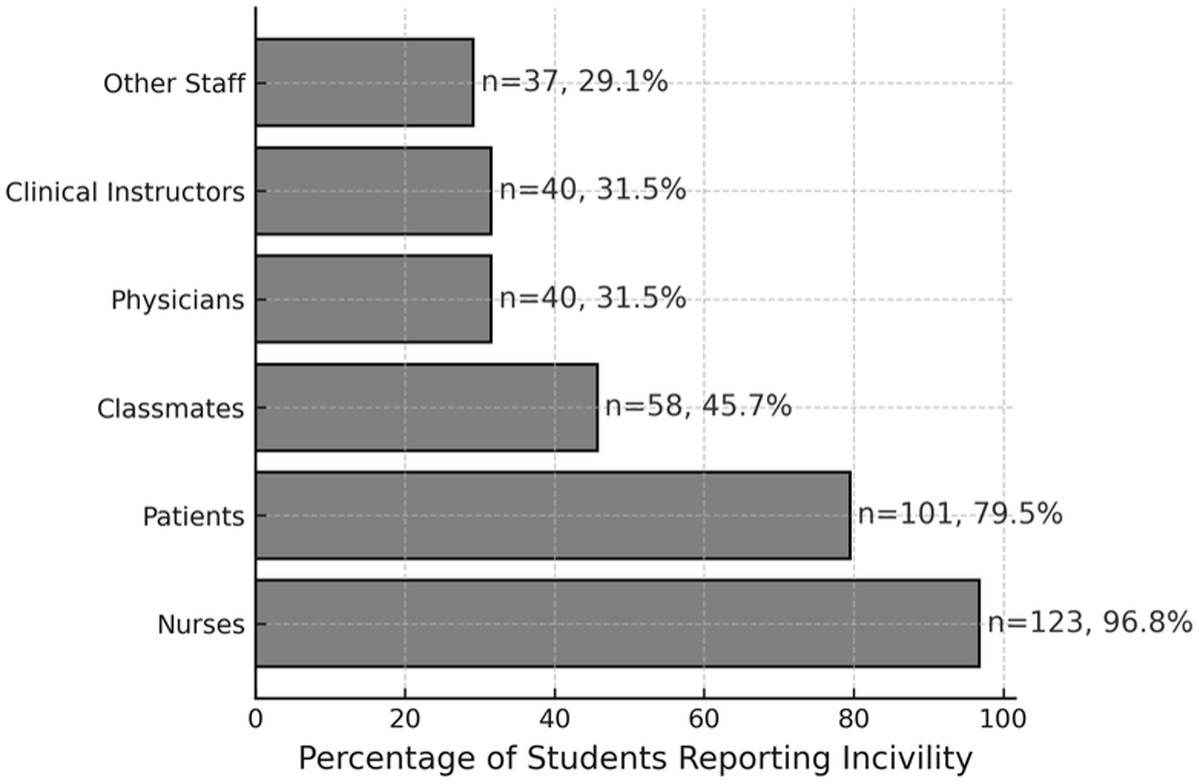
Distribution of clinical incivility experienced by nursing students (n = 127).

Sample characteristics (N = 159) as revealed in the participant characteristics survey are displayed in Table 1. Participants’ average age was 22.89 years (SD = 2.22). Most reported no jobs (n = 124, 78.0%) and no religion (n = 97, 61.0%). A total of 127 nursing students (79.9%) reported experiencing clinical incivility during their clinical practice from nurses (n = 123, 96.8%), patients (n = 101, 79.5%), classmates (n = 58, 45,7%), clinical instructors (n = 40, 31.5%), physicians (n = 40, 31.5%), and other staff (n = 37, 29.1%; see Fig 1). For subsequent survey data analysis, the participants were divided into a Clinical Incivility group (n = 127) and a No Clinical Incivility group (n = 32).

**Table 1:** Sample characteristics (N = 159).

Variable		Value
Age, *M (SD)*		22.89 (SD = 2.22)
Marital Status, *n (%)*		
	Single/With no partner	154 (96.9)
	Married/Living with partner	5 (3.1)
Education, *n (%)*		
	High School Diploma	146 (91.8)
	Bachelor’s Degree or Higher	13 (8.2)
Employment, *n (%)*		
	Unemployed	124 (78.0)
	Employed (Full-time & Part-time)	35 (22.0)
Religion, *n (%)*		
	Protestant	40 (25.2)
	Catholic	12 (7.5)
	Buddhis	10 (6.3)
	No religion	97 (61.0)

Clinical incivility from nurses experienced by the nursing students was further measured by the K-UBCNE. Interestingly, the group that experienced incivility reported significantly higher levels of Exclusion (t = 2.483, p = .014), Contempt (t = 4.433, p < .001), and Refusal (t = 2.053, p = .042) from nurses compared to those who did not experience incivility (See Table 2). The mean total K-UBCNE score for the Clinical Incivility group (37.03 ± 11.73) was notably higher than that of the No Clinical Incivility group (28.91 ± 12.38), with this difference being statistically significant (t = −3.53, p < .001). These results indicate that nursing students who had experienced clinical incivility encountered, as a group, higher levels of specific uncivil behaviors than the students who reported not having experienced clinical incivility.

**Table 2:** t-test results comparing clinical incivility and no clinical incivility groups.

Variables (K-UBCNE)	Clinical Incivility Group (n = 127)		No Clinical Incivility Group (n = 32)		*t*(157)	p	Cohen’s *d*
	*M*	*SD*	*M*	*SD*			
Exclusion	14.614	5.162	12.031	5.631	2.483	.014	5.258
Contempt	15.511	5.087	11.062	5.015	4.433	.001	5.073
Refusal	6.905	2.706	5.812	2.632	2.053	.042	2.691

Note. K-UBCNE = Korean Version-Uncivil Behavior in Clinical Nursing Education; M = Mean; SD = Standard Deviation

The mean score of stress levels measured by the K-PSS in the Clinical Incivility group (29.90 ± 4.10) was higher than that of the No Clinical Incivility group (26.81 ± 3.59); the difference between scores was statistically significant (t = −4.44, p < .001). By contrast, there was no statistically significant difference between the two groups’ ratings of BSN Program Satisfaction, Clinical Practice Satisfaction, and Clinical Site Satisfaction, the three items in the BSN Clinical Practice Evaluation Survey.

Use of the chi-square statistic for categorical variables including marital status, education, employment, and religion revealed no significant difference between the two groups on these characteristics. This means that the distribution of these categorical variables was similar in both groups; any observed differences were not statistically significant.

**Fig 2:**
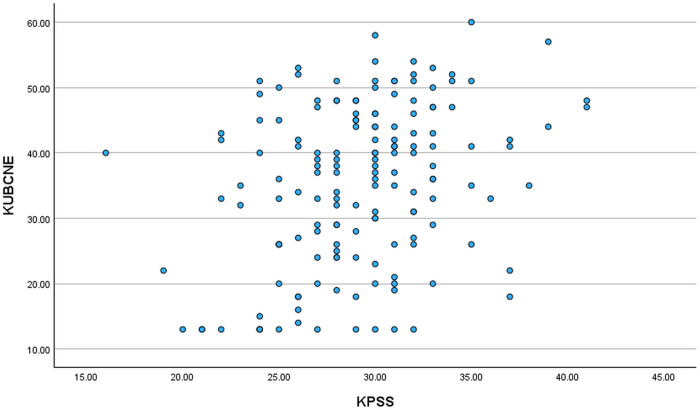
A scatterplot of correlation between clinical incivility and stress. *Note*: K-UBCNE = Korean Version-Uncivil Behavior in Clinical Nursing Education; K-PSS = Korean Version-Perceived Stress Scale.

Pearson correlations were calculated among the following variables: age, GPA, K-UBCNE total score, K-PSS total score, and BSN Program Satisfaction, Clinical Practice Satisfaction, and Clinical Site Satisfaction scores (See Table 3). The total score of the K-UBCNE was positively correlated with the total score of the K-PSS (r = .317, p < .001), indicating that participants who reported more experiences of incivility also reported higher levels of stress (See Fig 2). On the other hand, the total score of the K-UBCNE was negatively correlated with Clinical Practice Satisfaction (r = −.251, p < .001) and Clinical Site Satisfaction (r = −.375, p < .001). This means that as levels of incivility, as measured by the K-UBCNE, increased, satisfaction with clinical practice and the clinical site decreased. In other words, greater experiences of incivility were associated with lower satisfaction levels in both clinical practice and the clinical site.

**Table 3:** Intercorrelations among the study variables.

	Age	GPA	K-UBCNE	K-PSS	BSN Program Satisfaction	Clinical Practice Satisfaction	Clinical Site Satisfaction
Age	1						
GPA	.138	1					
K-UBCNE	.185	.193	1				
K-PSS	.017	.194	.317***	1			
BSN Program Satisfaction	.123	.207	.119	.293	1		
Clinical Practice Satisfaction	−.043	.060	−.251***	−.053	.446***	1	
Clinical Site Satisfaction	−.032	.037	−.375***	−.059	.298***	.799***	1

*Note*: GPA = Grade Point Average; K-UBCNE = Korean Version-Uncivil Behavior in Clinical Nursing Education; K-PSS = Korean Version-Perceived Stress Scale; BSN = Bachelor of Science in Nursing

* p < .05 (2-tailed), ** p < .01 (2-tailed), *** *p* < .001 (2 –tailed).

### 3.2. Qualitative data

Twenty nursing students participated in the focus group sessions. Analysis of the sessions’ transcripts yielded four themes and 10 subthemes (See Table 4). The four themes were: 1) *Being defenselessly exposed to verbal or nonverbal abuse*, 2) *Not being treated as a student nurse*, 3) *Experiencing a combination of negative feelings*, and 4) *Taking incivility for granted in the clinical site.*

**Table 4:** Themes and subthemes of clinical incivility experienced by nursing students.

Themes	Subthemes
1. Being defenselessly exposed to verbal or nonverbal abuse	- Hurtful and rude words or actions - Yelling and scolding - Not being called by name
2. Not being treated as a student nurse	- Ignored - Lack of nurses’ support for students’ clinical education - Pressure to conduct nurses’ troublesome or unpleasant work
3. Experiencing a combination of negative feelings	- Embarrassment - Coexistence of anger and sadness - Fear of being scolded - Worry about the future
4. Taking incivility for granted in clinical sites	- Overworked status of nurses in clinical sites - Lack of nurses’ awareness of clinical incivility - Nurses’ work in environments where incivility is passed down

#### 3.2.1. Being defenselessly exposed to verbal or nonverbal abuse.

**Hurtful and rude words or actions:** Students described encountering both verbal and nonverbal abuse from the nurses throughout their clinical experiences. Nurses often exhibited frustration when responding to students’ questions, which was reflected in their dismissive body language and sharp tone. This behavior included sighing heavily, rolling their eyes, or making sarcastic comments, all of which contributed to a climate of intimidation. On multiple occasions, the students overheard nurses publicly comparing them to their peers in a disparaging manner. Additionally, some nurses threatened students whom they disliked, warning them not to even consider applying for a job at the hospital where they worked.

*Student AJ: The nurse said, “Why did you come to the clinical practice without studying?” (sighing heavily and rolling her eyes) After that, I had an incivility experience that made me feel bad because the nurse kept saying things that put me down like that.*

*Student KR: The nurses gathered to compare the nursing students with former students, saying, “These nursing students seem to be lazier than last week’s students. Look how they did.”*

*Student JJ: When I wasn’t performing well during my clinical practice, some nurses would say hurtful things and then just walk away, which made me feel disrespected.*

**Yelling and scolding:** Students felt harshly scolded by the nurses for small mistakes, reporting being mistakenly scolded even when they hadn’t done anything wrong and in public places without waiting to hear an explanation. One student described a nurse scolding her for asking the ward doctor in detail about patient care.

*Student SS: The nurse yelled at me, “What did you do? It’s wrong.” And she screamed, “You’re annoying.”*

*Student KS: The nurse yelled at me and said, “Why are you asking the doctor? You’re here to help the nurses. Come on, help the nurses!”*

*Student GR: One day, I made a mistake and took a long time measuring vital signs. A nurse yelled and scolded me, saying, “How can you still be struggling with something like that?‘It was very distressing.*

**Not being called by name:** The nurses often used informal language when calling the nursing students, saying “*Hey*” instead of using their names or simply saying, “*Student Nurses*.” The students expressed feelings of offense and disrespect when addressed informally and unprofessionally, without being called by name, which made them feel devalued.

*Student RI: The nurse told us, “Hey, you come here.” However, I didn’t know who the nurse was asking because I was with the other students. Nevertheless, since we were in a public place and had a professional relationship, I wondered if addressing me as “Hey” was appropriate.*

*Student JJ: During the clinical, the nurse called out to me in an angry voice, “Hey, you, student”. At that moment, I felt disrespected and demoralized by the way the nurse addressed me. Being referred to as “you, student” instead of by my name made me feel devalued and unappreciated as a nursing student.*

#### 3.2.2. Not being treated as a student nurse.

**Ignored:** The nursing students felt ignored during their clinical practice. Specifically, the nurses disregarded the students’ greetings and dismissed their knowledge and skills. Some nurses would even say things like, “*You guys know nothing*!”, “*What do you know*?”, and “*How much could you really do*?” without providing proper feedback on the students’ performance. The students also mentioned that nurses acted as if the students didn’t exist, offering no communication or response, leaving the students to endure an uncomfortable and tense atmosphere.

*Student KS: The nurses scolded us if we didn’t say hello. However, when we said hello to them, they ignored our greeting. I was confused. I think the nurses ignored the nursing students.*

*Student SK: Whenever I tried to carry out the nursing practice I learned at school, the nurse didn’t let me. However, I did not know what I had done wrong.*

*Student AJ: On my first day of clinical, I followed a nurse to perform nursing skills, but she didn’t communicate with me or offer any meaningful feedback. It felt like I wasn’t even there, which made me very uncomfortable.*

**Lack of nurses’ support for students’ clinical education:** The students reported that nurses at clinical sites frequently provided minimal support for their clinical education. Some nurses were either unprepared or unwilling to engage in teaching roles, resulting in missed opportunities for hands-on learning and guidance. As students followed nurses to observe and practice their knowledge and skills, they encountered significant reluctance or outright refusal from nurses to participate in the educational process. For example, the students observed that their orientation and instruction on clinical wards were either poorly managed or entirely omitted, further diminishing the essential support required for their education.

*Student KR: I was following the nurses to observe nursing care. However, one nurse looked back at me and said, “Don’t follow me. I don’t need a nursing student.”*

*Student HS: The nurses on the clinical wards showed no interest in supporting nursing students. We received no assistance, and essential resources such as rest areas, lab materials, and relevant information were not provided, leaving us unsure of how to proceed.*

*Student GR: In a ward, I followed a nurse around all day to observe, but there was no specific instruction or orientation provided. I began to question whether this was truly considered clinical practice and education.*

**Pressure to conduct nurses’ troublesome or unpleasant work:** Nursing students reported feeling that they were often misused to handle nurses’ troublesome and unpleasant tasks, rather than being given the opportunity to gain experience in performing the full range of nursing care. As a result, they found it difficult to voice their concerns when placed in uncomfortable or undesirable situations. Some students expressed discomfort when asked to assist with nurses’ tasks even after their clinical hours had ended due to the busy environment. They felt that, rather than being treated as nursing students in clinical practice, they were viewed as task handlers, deprived of the respect and learning opportunities they deserved.

*Student IC: I went around all day helping with patients’ miscellaneous needs and patient transfers rather than holistic professional nursing care. As a nursing student, I came to the hospital for hands-on healthcare practice, but when this occurs frequently, I begin to question what I’m actually learning and what I should be gaining from my clinical experience. It makes me uncertain whether I’m receiving the proper clinical training.*

*Student TT: The nurses usually let the nursing students deal with demanding patients in the clinical wards. The demanding patients always made strange requests, so we, the nursing students, often faced embarrassing and uncomfortable situations. Despite this, the nurses, without considering our circumstances, routinely delegated tasks they found tedious or troublesome. Unable to refuse, we carried out these tasks despite feeling uncomfortable. As a result, many students expressed that clinical practice became increasingly difficult each time this occurred.*

#### 3.2.3. Experiencing a combination of negative feelings.

**Embarrassment:** The nursing students reported that embarrassment is the initial reaction when they are subjected to disrespectful words or behaviors during clinical practice. Many noted that this embarrassment quickly turned into feelings of shame and discomfort, leaving them unsure of how to respond appropriately. The impact was even more profound when the disrespect occurred unexpectedly or in front of others, intensifying their humiliation. This experience triggered a range of negative feelings, including a significant decline in self-confidence, which further impaired their ability to fully engage in the learning process.

*Student BS: In that situation, I was very embarrassed at first. And I wondered why I should be treated badly like this. I did not know how to handle it.*

*Student SS: At first, I felt extremely embarrassed, especially with other nursing students and patients around, which made it even more humiliating, and I didn’t know what to do. Then, feelings of discomfort and negativity started to overwhelm me.*

*Student KS: I was overwhelmed with embarrassment, and then I became so negative, exhausted, and fearful that I was emotionally unable to focus on clinical care for a while.*

**Coexistence of anger and sadness:** The nursing students reported feeling angry with the nurses who scolded them for doing nothing wrong. Furthermore, the students were angry with themselves because they couldn’t do anything. At the same time, they felt unfairly treated; they couldn’t handle the uncivil situations and had to endure them alone.

*Student AR: The nurse was angry with me. However, it was an unfair situation. I was upset and wanted to explain what happened. But I didn’t know what to do. I only talked about the situation with my classmates, and I was sad.*

*Student RI: I was angry and frustrated because I didn’t know how to respond to the nurses’ rude behaviors and felt that I had no choice but to endure it. Later, I became saddened by my own situation.*

*Student SS: Even after finishing my clinical practice and going home, the experiences of rudeness continued to anger, distress, and sadden me.*

**Fear of being scolded:** The nursing students were scolded by the nurses for minor mistakes, asking questions, and other trivial things. As a result, they were afraid of being scolded and felt the need to gauge the nurses’ emotions throughout the clinical.

*Student IC: I became cautious no matter what I did and worried that I would do something wrong. I was always careful not to be scolded.*

*Student AJ: The rude words and behaviors I encountered during my clinical sites made me overly cautious and anxious, constantly fearing being scolded by a nurse.*

*Student SS: When I made a mistake, my nurse was rude and made the situation very difficult for me, causing me to fear being scolded again for any future errors.*

**Worry about the future:** The nursing students’ incivility experience triggered worry about becoming future RNs. They wondered whether they would perform their duties well as future nurses, stating they seriously reconsidered pursuing nursing, fearing that they would not be able to do well in their daily and work lives in the future.

*Student AR: I reconsidered being a nurse for my job.*

*Student RP: If I become a nurse and experience such uncivil words or behaviors later, I wonder how I should deal with it. I doubted whether I should be a nurse or not.*

*Student SK: The rudeness experienced during clinical practice had a significantly negative impact on me, and I became fearful of working as a nurse in a hospital. The thought of being treated with such disrespect as a new graduate nurse shattered my illusions about the profession and led me to question whether choosing a nursing career was the right decision.*

#### 3.2.4. Taking incivility for granted in clinical sites.

**Overworked status of nurses in clinical sites:** The major cause of clinical incivility the nursing students described was the excessive workload of the nurses. The nurses worked busily throughout their working hours due to their large patient assignments and heavy workload. The nurses had to finish their work on time, thus were sensitive to tasks and emergencies that might arise.

*Student TT: The nurses’ heavy work forced them to become more sensitive, which I think eventually led to rude words or actions to the nursing students*.

*Student IC: First of all, most nurses seemed to have an overwhelming workload. New graduate nurses, in particular, often appeared to be struggling. Because of this, it likely wasn’t easy for them to help nursing students, and it may have felt like a burden, making it difficult to even ask for their help.*

*Students JJ: In some clinical wards, the nurses always seemed busy. Seeing how difficult their work was, I felt that it wouldn’t be easy for them to help us, and as a result, I thought that the nurses might become more frustrated and irritated with the students.*

**Lack of nurses’ awareness of clinical incivility:** The students noted that no nurse took incivility seriously during the clinical; they reported they knew no adequate responses to use with nurses who treated nursing students or fellow nurses rudely. The nursing students also said that incivility seems to be taken for granted and ignored because the structure of the nursing profession is a vertical culture rather than a horizontal culture.

*Student AG: The nurses seem to take it for granted that they are rude to the nursing students.*

*Student AJ: There’s also widespread disrespect among the nurses.*

*Student BS: The nurse who scolded the nursing student also displayed rude behavior and language toward other fellow nurses. This made me realize that incivility in the clinical setting is not just an issue between nurses and nursing students.*

**Nurses work in environments where incivility is passed down:** The students observed that the culture of rudeness among nurses is being passed down. They described a situation where a nurse, after experiencing incivility from a senior nurse, treated a newly graduated nurse even more harshly. In turn, the newly graduated nurse mistreated the nursing students.

*Student RP: I didn’t feel good because the newly graduated nurse who got scolded by a senior nurse seemed to be taking it out to the nursing students.*

*Student JY: I think this disrespect is widespread among nurses and continues to be passed on to nurses.*

*Student KS: I noticed that the newly graduated nurse, who was treated rudely by the senior nurse, was repeating the same behavior to us. It felt like the incivility was being passed down, and I started to worry that I might end up doing the same when I become a nurse.*

## 4. Discussion

Our mixed methods study was conducted to investigate the relationship between clinical incivility experienced by pre-licensure nursing students and their stress levels as well as to better understand students’ personal experiences of clinical incivility received from nurses.

Experience of clinical incivility was reported by 79.9% (n = 127) of the Korean nursing students in our study. Kim et al. [35] and Kim et al. [39] reported the prevalence of clinical incivility among other Korean nursing student samples was 91.46% (n = 375 out of 410) and 73.05% (n = 122 out of 167) respectively; major perpetrators were nurses at the clinical sites. In the study by Minton et al. [55], the prevalence of clinical incivility experienced by participating New Zealand nursing students was 53% (n = 149 out of 281); nurses were also identified as the primary perpetrators. Nurses play a vital role in helping students learn and practice nursing knowledge and skills. However, many nurses give nursing students a difficult time in the clinical environment.

In our study of 159 Korean nursing students, the correlation between clinical incivility (measured by K-UBCNE) and stress (measured by K-PSS) (r = .317, p < .00) mirrored the positive correlation observed between incivility scores on the UBCNE and reported stress levels on the PSS among 35 U.S. nursing students in a prelicensure BSN (PL-BSN) program (r = .484, p = .003) [56]. This reaffirms that experiencing incivility during clinical practice is associated with increased stress among nursing students. Clinical practice in a new environment where nursing students interact with real patients is likely to cause stress in nursing students even absent incivility experiences. Such stress has been linked to lower self-efficacy, reduced life satisfaction, lower life orientation, and decreased self-esteem, as shown in Bodys-Cupak’s 2019 study of 307 nursing students in Poland [57]. The nursing students in our study who experienced more clinical incivility also reported higher levels of stress. Greater clinical incivility experience also significantly correlated with lower Clinical Practice Satisfaction (r = −.251, p < .001) and lower Clinical Site Satisfaction (r = −.375, p < .001). This underscores the need to address incivility to reduce stress and improve satisfaction with both the clinical practice environment and students’ clinical experiences, crucial for achieving positive clinical education outcomes.

In the Phase Two focus group sessions, nursing students described uncivil words and actions they experienced from the nurses during their clinical practice. The students felt ‘*Being defenselessly exposed to verbal or nonverbal abuse*’ and as if they were ‘*Not being treated as a student nurse’*. Participating students felt disrespected by the nurses, often not being called by name or being the butt of offensive and belittling remarks; in serious cases, the nurses yelled at the students or severely scolded them in public places. These findings are consistent with those of other studies conducted on nursing students [25,58,59].

In general, nursing students are under a lot of stress due to the new clinical environment, new people, and new patient care practices and systems in clinical sites [60]. Nursing students need attention, support, and encouragement to complete their clinical practicums. Rude words or actions by nurses can increase nursing students’ mental distress and stress levels [39]. Nurses’ use of rude language is related to their communication competence [61]. To reduce nurses’ disrespect to nursing students, it is necessary to develop interventions to improve their professional communication skills. Nurses must first acknowledge and actively address the ongoing issue of incivility toward students in clinical practice, recognizing how effective communication can promote collaborative interactions and create a more supportive learning environment. Furthermore, building camaraderie, practicing empathy, and engaging in active listening can enhance communication. These efforts will cultivate trust and mutual respect between nurses and nursing students, leading to greater understanding and reducing instances of incivility in the clinical setting [62].

Our study revealed that students did not always receive clear instructions as student nurses at the practice sites, nor were they always provided with appropriate seating and rest areas. Clinical practicums should afford students opportunities to practice what they have learned in the classroom and campus skills laboratory, but students in our study reported not only not being helped by the nurses in the clinical wards but being denied important opportunities to learn nurses’ roles and responsibilities. The nurses considered the nursing students as labor assistants who came to the clinical units to help them rather than as learners. Focus group members reported that miscellaneous work other than nursing care activities, such as checking for patients’ lost items or moving patients to other wards, were assigned to them rather than professional nursing activities they needed to learn. Similar themes were found in previous studies on nursing students who experienced clinical incivility including *‘students excluded from nursing fields as burdensome entities’, ‘experiencing limits in practical training’, ‘performing tasks irrelevant to the purpose of the training’, and ‘excessive work performance’* [25,58]. These experiences can be related to ‘*nurses’ lack of awareness of clinical incivility among nursing students, lack of awareness of the importance of student instruction during clinical’,* and ‘*lack of content or methods of student instruction during the clinical’* [63]. Nurse managers or administrators in clinical settings must consider whether the hospital environment is well-prepared to educate nursing students. They should also assign qualified professional nurses to act as preceptors for the students and collaborate with faculty to ensure that the students’ clinical experience is a productive learning opportunity. This approach is not only aimed at improving nursing practice education but also serves as a method to achieve the positive outcome of enhancing the quality of healthcare through the development of well-educated future nurses. Clinical incivility negatively affects nursing professional values [30,64] and the turnover intention of nurses [65]. Interventions are needed that promote nursing students’ retention in the profession. Upon conducting a quasi-experimental study, Kim et al [41] found an interactive clinical incivility management program helped PL-BSN nursing students gain needed knowledge to professionally handle incivility during clinical practice. Based on these findings, offering education on managing incivility to new nursing students before their clinical rotations is recommended.

Kim et al. [39] and Hong et al. [66] found that the greater the nurses’ clinical incivility to students, the more stress the nursing students felt during the clinical practicum. Our study also found a statistically significant positive correlation between experience of clinical incivility and stress levels. In the focus group sessions participants reported ‘*Experiencing a combination of negative feelings’* such as embarrassment, anger, sadness, and fear of getting scolded in addition to stress from their experiences of clinical incivility. These negative emotions affect students’ motivation and willingness to learn in clinical settings and amplify questions about the purpose and reason for clinical practice [67]. Negative emotions affect the process of becoming a nurse, a process that is eased by supporting positive thinking such as career hope, optimism, and resilience [68]. Students in our focus groups who experienced clinical incivility prospectively imagined the potential disrespect they might face as future nurses; they were fearful and found themselves questioning whether they should pursue a nursing career.

The theme, ‘*Taking incivility for granted in the clinical sites*’ related to a sub-theme explaining that theme, the ‘*Overworked status of nurses in clinical sites*’. Title 22 of the California Code of Regulations, Section 70217(a), effective January 1, 2008, requires that hospitals always have a nurse-to-patient ratio of 1:4 or less [69]. Meanwhile, according to data regarding nursing services in 2016 [70], the nurse-to-patient ratio of hospitals in Korea varied from 1:7 to 1:12. Korean nurses’ workload was relatively excessive. In addition, full-time nurses in the US typically work 36–40 hours per week [71], whereas nurses in Korean hospitals work about 48 hours per week, often skipping meals and delaying using the restroom to manage their work [72]. It was also revealed that 37% of Korean nurses’ working time was spent on direct nursing activities such as vital signs and health assessment [73]. This number is lower than 54% of direct nursing time in the USA [74], which indicates also that the hours of direct nursing care are limited due to the high nurse to patient ratio.

The students in our study suggested clinical incivility continues to occur due to a ‘*Lack of nurses’ awareness of clinical incivility’*. The seriousness of nurses’ clinical incivility has been on the rise in Korea, but there is a dearth of clinical incivility education and prevention programs for both nurses and nursing students. There is often no systematic nursing school or clinical site policy for addressing or helping students manage clinical incivility; students are left to cope with the problem individually. Thus, it is vital for nursing schools and clinical agencies to collaborate in addressing clinical incivility. Nursing schools and clinical partners should discuss the impact of clinical incivility on nursing students and develop strategies for effective management. Policies should be developed to define incivility clearly, outline the responsibilities of all parties involved, and establish procedures for reporting and addressing incidents. In addition to providing counseling support for students experiencing mental distress and stress caused by incivility [25,30], it is crucial to implement upstream interventions to prevent and manage the issue more comprehensively. Clinical incivility is not confined to interactions between nurses and nursing students; it can occur across departments and systems within clinical agencies [75]. Our study underscores the need for policies and programs focused on monitoring, identifying, and intervening in clinical incivility to reduce its negative effects on nursing students, nurses, other healthcare providers, and ultimately, patients [64].

### 4.1. Limitations

Our study findings can help educators and nurses better understand nursing students’ uncivil experiences during clinical practice and inform nursing schools and healthcare organizations of the need to develop programs to prevent and intervene in clinical incivility. However, there are limitations to the study. Our study sample, which consisted of nursing students from a single region in South Korea, limits the generalizability of the findings to other countries or regions with different cultural and healthcare practices. Additionally, participants may have felt hesitant to share negative experiences with each other during focus group sessions. To better support participants in fully expressing their experiences, particularly unpleasant or hurtful ones, future studies should consider conducting individual interviews instead of focus groups. Our study explored nursing students’ recent experiences and feelings about incivility, but the cross-sectional design limited our ability to assess changes over time. Future research using a longitudinal design is warranted, as it would provide deeper insights into how clinical incivility impacts stress and professional development throughout students’ training. Furthermore, our study focused exclusively on nursing students. Future research could broaden this focus to include students from other health sciences whose education includes clinical field experiences.

## 5. Conclusions

Many students experience mental distress from clinical incivility and associated stress. Students try to overcome this distress, often on their own, while simultaneously managing the challenges of nursing studies and their concerns about future work as a nurse. Clinical practicums are for nursing students; helping them learn during practicums is the responsibility of both nurse educators and nurses employed in clinical settings [76]. More effort should be invested in the development of evidence-based clinical incivility interventions or programs. These efforts have the potential to support students’ mental health and learning, increasing the probability they will remain in the profession.

## Supporting information

S1 File Dataset. (XLSX)
